# Analysis of cDNA libraries from developing seeds of guar (*Cyamopsis tetragonoloba *(L.) Taub)

**DOI:** 10.1186/1471-2229-7-62

**Published:** 2007-11-23

**Authors:** Marina Naoumkina, Ivone Torres-Jerez, Stacy Allen, Ji He, Patrick X Zhao, Richard A Dixon, Gregory D May

**Affiliations:** 1 Plant Biology Division, The Samuel Roberts Noble Foundation, 2510 Sam Noble Parkway, Ardmore, Oklahoma 73401, USA; 2 National Center for Genome Resources, 2935 Rodeo Park Drive East, Santa Fe, New Mexico 87505, USA

## Abstract

**Background:**

Guar, *Cyamopsis tetragonoloba *(L.) Taub, is a member of the *Leguminosae *(*Fabaceae*) family and is economically the most important of the four species in the genus. The endosperm of guar seed is a rich source of mucilage or gum, which forms a viscous gel in cold water, and is used as an emulsifier, thickener and stabilizer in a wide range of foods and industrial applications. Guar gum is a galactomannan, consisting of a linear (1→4)-β-linked D-mannan backbone with single-unit, (1→6)-linked, α-D-galactopyranosyl side chains. To better understand regulation of guar seed development and galactomannan metabolism we created cDNA libraries and a resulting EST dataset from different developmental stages of guar seeds.

**Results:**

A database of 16,476 guar seed ESTs was constructed, with 8,163 and 8,313 ESTs derived from cDNA libraries I and II, respectively. Library I was constructed from seeds at an early developmental stage (15–25 days after flowering, DAF), and library II from seeds at 30–40 DAF. Quite different sets of genes were represented in these two libraries. Approximately 27% of the clones were not similar to known sequences, suggesting that these ESTs represent novel genes or may represent non-coding RNA. The high flux of energy into carbohydrate and storage protein synthesis in guar seeds was reflected by a high representation of genes annotated as involved in signal transduction, carbohydrate metabolism, chaperone and proteolytic processes, and translation and ribosome structure. Guar unigenes involved in galactomannan metabolism were identified. Among the seed storage proteins, the most abundant contig represented a conglutin accounting for 3.7% of the total ESTs from both libraries.

**Conclusion:**

The present EST collection and its annotation provide a resource for understanding guar seed biology and galactomannan metabolism.

## Background

Guar, or clusterbean (*Cyamopsis tetragonoloba *(L.) Taub), is a drought-tolerant annual legume, which originated in the India-Pakistan area, and was introduced into the United States in 1903 [1]. Unlike the seeds of other legumes, guar seeds have a large endosperm, accounting for 42% of seed weight [2]. The predominant portion of the endosperm is mucilage or gum (guar gum), which forms a viscous gel in cold water. Approximately 80–85% of the gum is a galactomannan, consisting of a linear (1→4)-β-linked D-mannan backbone with single-unit, (1→6)-linked, α-D-galactopyranosyl side chains [3-6]. The galactomannan is in the form of non-ionic polydisperse rod-shaped polymers consisting of about 10,000 residues, which accumulate in the primary cell walls of the endosperm [7].

Galactomannans from various leguminous species have different degrees of galactose substitution. Low galactose galactomannans (25–35% galactose substitution) are typical for the more distantly related *Caesalpinoideae *sub-family of the *Leguminosae*, whereas higher degrees of galactose substitution (up to 97% in the tribe *Trifolieae*) are characteristic of the more closely related *Papilionoideae *legume sub-family [8]. Guar galactomannan has a mannose to galactose (M:G) ratio of 1.6 [5]. Pure mannan without galactose is completely insoluble in water, and increasing galactose substitution increases the solubility of the polymer by allowing it to become extended [9-11].

Galactomannans are multifunctional, assisting in water imbibition and drought avoidance before and during germination, and as a source of storage carbohydrate for the developing seedling [12]. Guar galactomannans form water dispersible hydrocolloids, which thicken when dissolved in water. Guar gum is therefore used as an emulsifying, thickening or stabilizing agent in a wide range of processed foods; as a stabilizer in ice cream and cake; to bind meat; and as a thickener in salad dressings and beverages [13]. Lower-grade guar gum has numerous industrial applications as a friction-reducing agent, for example in the manufacture of cloth and paper, in the petroleum industry, and in ore flotation.

Guar is economically the most important of the four species in the genus *Cyamopsis *[1]. Many publications over the past 60 years have described the properties of galactomannans and the food benefits of guar gum. However, despite the importance of the species, only a single report exists of the development of genomic resources in guar [14]. In this report the guar mannan synthase gene was identified from an expressed sequence tag (EST) collection derived from RNA isolated from guar seeds at three different stages of development, although no further details were given of the other EST sequences obtained. We here describe the features of an additional EST dataset derived from single pass sequencing of cDNAs of developing guar seeds. This should prove valuable for the understanding of seed-specific gene expression, by providing an extensive resource for the cloning of genes, development of markers for map-based cloning, and annotation of future genomic sequence information. The cloning of genes encoding enzymes of specific biochemical pathways by EST sequencing has been a very successful strategy, particularly when the cDNA libraries were prepared from specialized tissues with high activity for the respective enzymes [15,16]. ESTs and their accompanying cDNAs also provide the means to construct inexpensive macroarrays or microarrays, which can be used to study the expression of genes on a genome-wide scale [17,18]. Furthermore, within statistical limitations [19], the abundance of a specific cDNA in the EST collection is a measure of gene expression level. Using this premise, we present a preliminary evaluation of the expression patterns of sets of genes with different functional ontologies, particularly those potentially involved in storage polysaccharide and storage protein metabolism, during the development of guar seeds.

## Results and Discussion

### Generation of cDNA libraries

**Figure 1:**
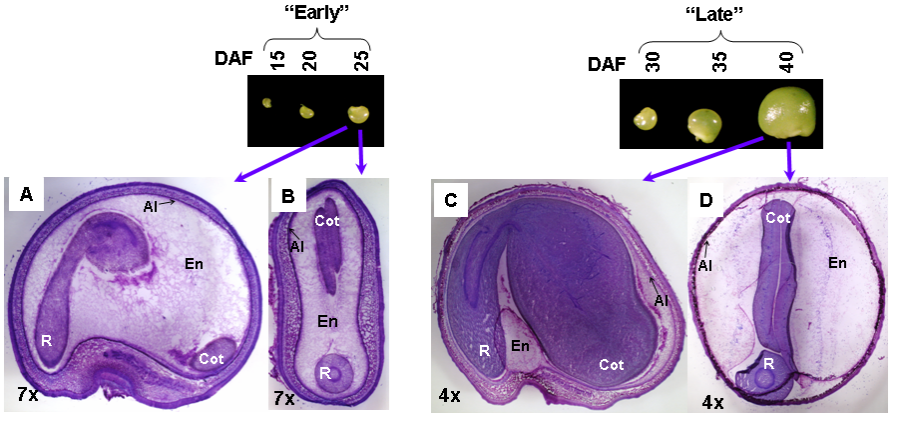
**Sections of guar seeds stained with toluidine blue**. (A)15 μm longitudinal section and (B) cross section (x7) of guar seed at 25 DAF; (C) longitudinal section and (D) cross section (x4) of guar seed at 40 DAF stained with toluidine blue 0.05%. Al, aleurone layer; Cot, cotyledon; En, endosperm; R, root.

**Figure 2:**
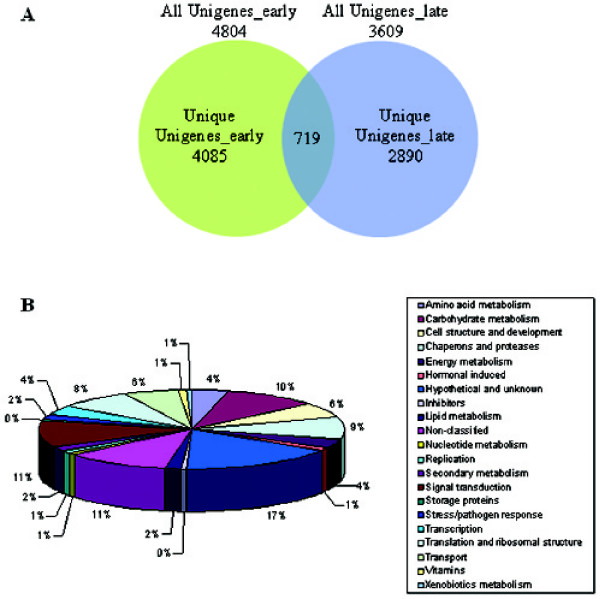
**Gene expression patterns based on EST counts**. (A) Venn diagram of unigenes detected in the "Early" (15–25 DAF) and "Late" (30–40 DAF) guar cDNA libraries. (B) Distribution of unigenes from the "secure" assignment category in classes of putative function. The classes of putative gene functions are presented in alphabetical order based on the description of the best match from BLASTX similarity searches to the non-redundant GenBank protein databases. (C) Comparison of EST numbers in the "early" and "late" development stage cDNA libraries, distributed into classes of putative function.

Figure 1 shows sections of developing guar seeds at 25 days after flowering (DAF) and of mature seeds at 40 DAF. The mature seeds have a large endosperm packed with reserves of carbohydrate (principally galactomannan), protein, lipid and minerals, which provide a reserve for the developing seedling for several days. In order to investigate developmentally regulated genes with a focus on galactomannan biosynthesis, two cDNA libraries were constructed. The "Early" cDNA library (library I) was made from seeds 15, 20 and 25 DAF, and the "Late" library (library II) from seeds at 30, 35 and 40 DAF. Developmental time points (DAF) were chosen for pooling based on maximal transcript levels of two key enzymes of galactomannan biosynthesis, galactosyl transferase and mannan synthase [4,14,20]. As described in our results below, the highest expression level of galactosyl transferase was detected by RT-PCR at 35 DAF and no mannan synthase expression was detected prior to 30 DAF. In total 16,476 ESTs from both cDNA libraries were sequenced, comprising 8,163 and 8,313 ESTs from libraries I and II, respectively. A total of 7,694 unique sequences, or unigenes (UG) were identified, of which 1,695 represented contigs and 5,999 represented singletons. Library I contained 4,804 unigenes, and library II contained 3,609. Surprisingly, only 719 unigenes were common to both libraries (Figure 2A). EST sequences of all clones are available at GenBank (Accessions EG974821 through EG991296).

### Annotation and functional classification of guar ESTs

ESTs were annotated with reference to gene function using the results of BLASTX comparisons with the GenBank non-redundant protein database (NR). EST sequences were grouped in three categories based on the "bit score" S' [21] of the aligned sequence segment with the top database hit after BLASTX comparison. The "secure" assignment group contains 1,662 unigenes (22% of the total) with the S' score value equal to or greater than 200; the "putative" assignment group contained 3,941 unigenes (51%) with the S' scores less than 200; the "no assignment" group contained 2,091 unigenes (27%) with no score. A BLASTX comparison of the 2,091 unigenes with no score was made against the *Medicago truncatula *genome v 1.0 [22], which resulted in an additional 377 annotations. For sequences that did not have BLASTX scores, no protein similar to the translation product was present in the public databases at the time of analysis. We therefore assume that approximately 27% of the clones in the seed database encode previously undescribed proteins or may represent non-coding RNA.

The largest group of ESTs fell into the "putative" assignment group. This group could reduce dramatically with additional efforts to improve the length of the sequencing reads and quality of the sequence data. For most of the analyses described, only the "secure" assignment group was considered for distributing genes into functional categories in order to gain a preliminary understanding of metabolic processes during guar seed development (Figure 2B,C). However, both "secure" and "putative" assignment groups were used to identify candidate genes for specific biochemical pathways.

### Energy flow in developing guar seeds

Seed development is genetically programmed and is associated with striking changes in metabolite levels. Differentiation occurs successively, starting with the maternal and followed by the filial organs, which later become highly specialized storage tissues. A complex regulatory network triggers initiation of seed maturation and corresponding accumulation of storage products. This includes transcriptional and physiological reprogramming mediated by sugar and hormone-responsive pathways [23,24].

Galactomannan and seed storage proteins accumulate to high amounts in mature guar seeds, representing 26–32% and 23–31% of the seed dry weight, respectively [25]. The biosynthesis of carbohydrate and storage proteins in guar seeds is probably preceded by increased transcriptional activity for these processes. Consistent with this hypothesis, the distribution of functional ontologies in the EST database (excluding unknown, hypothetical and non-classified genes) revealed major contributions from genes annotated as encoding proteins involved in signal transduction (10.9%), carbohydrate metabolism (10%), chaperone and proteolytic processes (9%), and translation and ribosomal structure (7.8%) (Figure 2B).

Mature seeds have very low metabolic activity, reflected by the lower representation of specific EST classes in library II. Genes annotated as involved in signal transduction were represented by four times as many ESTs, carbohydrate metabolism three times, chaperone and proteolytic activity 1.8 times, and translation and ribosomal structure 1.4 times, in library I compared to library II (Figure 2C, Additional file 1). However, three functional categories were represented by higher numbers of ESTs in library II. These include seed storage proteins (SSPs), and hormonal and stress/pathogen induced genes. SSPs accumulate to high levels during the late stages of seed development. Among the "stress/pathogen response" group of genes, one highly induced contig (UG00086) was represented by 46 ESTs in library II. This gene showed 81% amino acid similarity to a ripening-related protein from soybean (*Glycine max*) [GB# AAD50376] which is activated in soybean-soybean cyst nematode interactions and contains a conserved domain for the pathogenesis-related protein Bet v I family.

UG00177, in the hormone-inducible functional category, was represented by 26 ESTs in library II. The encoded protein showed 85% amino acid similarity to an auxin down-regulated gene from soybean [26], the function of which is yet to be determined. Five and seven ESTs" in libraries I and II, respectively, corresponded to genes involved in the biosynthesis of gibberellic acid (GA) (Additional file 1). Synthesis of GA in developing seeds is necessary to promote cell expansion [27].

### Galactomannan metabolism

**Figure 3:**
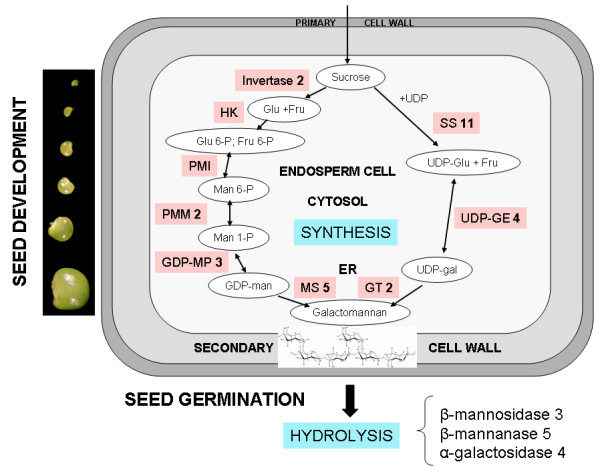
**Schematic representation of galactomannan metabolism in guar seeds**. This scheme was modified from [50]. Substrates are shown in white ovals, enzymes in pink rectangles. Numbers next to enzyme names correspond to the number of unigenes detected in the cDNA libraries (see Table 1 for details). Double-headed arrows indicate reversible reactions, single-headed arrows irreversible reactions. Abbreviations: Glu, glucose; Fru, fructose; Man, mannose; Gal, galactose; HXK, hexokinase; PMI, phosphomanno-isomerase; PMM, phosphomanno-mutase; GDP-MP, GDP-mannose pyrophosphorylase; MS, GDP-man-dependent mannosyl-transferase; GT, UDP-gal-dependent galactosyl transferase; SS, sucrose synthase; UDP-GE, UDP-galactose 4-epimerase.

*Biosynthesis *– Galactomannan is the major storage polysaccharide in guar seeds and accumulates in cell walls of the endosperm, accounting for up to 26–32% of the seed dry weight [25]. Figure 3 shows an outline of galactomannan metabolism in guar, highlighting the importance of sucrose as a building block. In most plant species carbon is transported as sucrose. Cleavage of the *O*-glycosidic bond between the glucose and fructose units of sucrose is catalyzed by invertase (EC 3.2.1.26) and sucrose synthase (EC 2.4.1.13) [28]. Invertase is a hydrolase, cleaving sucrose irreversibly into glucose and fructose, whereas sucrose synthase is a glycosyl transferase, converting sucrose in the presence of UDP into UDP-glucose and fructose. Two ESTs corresponding to different invertase unigenes were detected only in library I. Likewise, of the 11 unigenes corresponding to sucrose synthases, most were also represented by ESTs found in library I (Table 1).

**Table 1:** Guar unigenes potentially involved in galactomannan metabolism

**Unigene ID**	**Early**	**Late**	**NR Top Hit**	**NR Top Hit Description**	**e value**
**Sucrose hydrolyzing enzymes**					
GUAR_UG_02470	1	0	CAA76145	neutral invertase [Daucus carota]	9e-110
GUAR_UG_02964	1	0	P29001	acid invertase (acuolar invertase)	1e-103
GUAR_UG_05135	1	0	AAC28107	nodule-enhanced sucrose syn [P. sativum]	6e-107
GUAR_UG_03949	1	0	AAC28107	nodule-enhanced sucrose syn [P. sativum]	2e-093
GUAR_UG_04997	1	0	AAC28107	nodule-enhanced sucrose syn [P. sativum]	9e-083
GUAR_UG_00403	4	0	AAC28107	nodule-enhanced sucrose syn [P. sativum]	2e-012
GUAR_UG_00496	3	1	Q01390	sucrose synthase	1e-026
GUAR_UG_01704	1	0	AAC39323	sucrose synthase [Glycine max]	9e-069
GUAR_UG_05973	0	1	AAC39323	sucrose synthase [Glycine max]	2e-047
GUAR_UG_04679	1	0	CAB39757	sucrose synthase [Lotus corniculatus]	5e-066
GUAR_UG_02621	1	0	CAA49428	sucrose synthase [Vicia faba]	7e-047
GUAR_UG_00402	2	0	CAC32462	sucrose synthase isoform 3 [Pisum sativum]	4e-011
GUAR_UG_03411	1	0	AAR31210	sucrose-phosphate synthase [M. sativa]	2e-035
**Nucleotide-sugar interconversion enzymes**					
GUAR_UG_02815	1	0	CAA06338	UDP-galactose 4-epimerase [C. tetragonoloba]	2e-045
GUAR_UG_04018	1	0	Q43070	UDP-galactose 4-epimerase	2e-091
GUAR_UG_00429	3	3	XP_474395	phosphomannomutase [Oryza sativa]	2e-058
GUAR_UG_03026	1	0	XP_474395	phosphomannomutase [Oryza sativa]	2e-084
GUAR_UG_06634	0	1	BAB62108	GDP-D-mannose pyrophosphorylase	2e-039
GUAR_UG_02247	1	0	AAD22341	GDP-mannose pyrophosphorylase [Arab.]	7e-095
GUAR_UG_07483	0	1	AAN15442	GDP-mannose pyrophosphorylase [Arab.]	5e-029
**Glycosyl transferases**					
GUAR_UG_07564	0	1	AAR23313	β-1,4-mannan synthase [C. tetragonoloba]	2e-062
GUAR_UG_07598	0	1	AAK49454	cellulose synthase CesA [Nicotiana alata]	1e-036
GUAR_UG_04832	1	0	NP_197666	glycosyl transferase family 2 [Arabidopsis]	2e-037
GUAR_UG_04940	1	0	NP_181493	glycosyl transferase family 2 [Arabidopsis]	1e-022
GUAR_UG_02980	1	0	XP_473388	mannosyltransferase family [Oryza sativa]	3e-054
GUAR_UG_05797	0	1	CAI79402	galactosyltransferase [C. tetragonoloba]	4e-033
GUAR_UG_03477	1	0	BAD37266	galactosyltransferase [Oryza sativa]	4e-022
**Glycoside hydrolases**					
GUAR_UG_00260	10	1	CAC08442	(1–4)-β-mannan endohydrolase [C. arabica]	8e-047
GUAR_UG_03304	1	0	CAC08442	(1–4)-β-mannan endohydrolase [C. arabica]	1e-046
GUAR_UG_06736	0	1	CAC08442	(1–4)-β-mannan endohydrolase [C. arabica]	5e-005
GUAR_UG_01175	2	0	CAC51690	endo-β-1,4-mannanase [Lactuca sativa]	3e-008
GUAR_UG_00259	12	1	AAN34823	endo-β-mannanase [Daucus carota]	4e-019
GUAR_UG_00294	0	14	AAL37714	β-mannosidase enzyme [L. esculentum]	2e-073
GUAR_UG_05641	0	1	AAL37714	β-mannosidase enzyme [L. esculentum]	1e-057
GUAR_UG_06448	0	1	AAL37714	β-mannosidase enzyme [L. esculentum]	6e-079
GUAR_UG_02026	1	0	AAN32954	α-galactosidase [L. esculentum]	1e-007
GUAR_UG_03848	1	0	CAF34023	α-galactosidase 1 [Pisum sativum]	1e-045
GUAR_UG_05497	0	1	CAF34023	α-galactosidase 1 [Pisum sativum]	3e-089
GUAR_UG_02208	1	0	NP_189269	α-galactosidase [Arabidopsis]	2e-040
**Sugar transporters**					
GUAR_UG_03740	1	0	NP_849565	sugar transporter [Arabidopsis]	4e-041
GUAR_UG_02994	1	0	NP_180526	sugar transporter [Arabidopsis]	1e-072
GUAR_UG_01798	1	0	NP_180526	sugar transporter [Arabidopsis]	3e-052
GUAR_UG_04700	1	0	NP_850483	sugar transporter [Arabidopsis]	2e-079
GUAR_UG_04227	1	0	NP_850835	sugar transporter [Arabidopsis]	1e-056
GUAR_UG_02250	1	0	NP_174313	sugar transporter [Arabidopsis]	4e-049
GUAR_UG_00912	2	0	NP_174313	sugar transporter [Arabidopsis]	7e-015
GUAR_UG_02913	1	0	NP_567083	nucleotide-sugar transporter [Arabidopsis]	8e-072
GUAR_UG_03734	1	0	AAU07980	hexose transporter [Vitis vinifera]	2e-055
GUAR_UG_03820	1	0	AAB88879	sugar transporter [Prunus armeniaca]	2e-099
GUAR_UG_03654	1	0	AAT40483	UDP-galactose transporter [S. demissum]	7e-044
GUAR_UG_05960	0	1	CAD91334	sucrose transporter [Glycine max]	2e-010

During seed development, entry of carbon from the maternal coat cells into the seed apoplasm is mediated by membrane-localized sugar transporters [29,30]. Twelve unigenes annotated as sugar transporters were found in the guar seed cDNA libraries (Table 1). All ESTs, with the exception of UG05960, were detected in library I, suggesting that sugar transporters are actively transcribed, and presumably function, during early stages of guar seed development.

No hexokinase ESTs were detected in either of the cDNA libraries. Plant hexokinase (HXK) has been shown to be involved in sugar sensing and signalling, and is proposed to be a dual-function enzyme with both catalytic and regulatory functions [31-34]. For example, transgenic *Arabidopsis *plants over-expressing *AtHXK1 *and *AtHXK2 *showed enhanced sensitivity to glucose containing medium [31]. Overexpression of the Arabidopsis *AtHXK1 *in transgenic tomato plants led to reduced photosynthetic activity [32]. HXK is presumably encoded by low abundance transcripts in developing guar seeds.

Phosphomannoisomerase (EC 5.3.1.8) converts fructose-6-phosphate (Fru-6-P) to mannose-6-phosphate (Man-6-P). This enzyme also functions in the reverse direction in the utilization of mannose released by hydrolysis of galactomannan on germination, after it is phosphorylated to Man-6-P [35]. No ESTs annotated as phosphomannoisomerase were detected in either of the libraries. However, two unigenes corresponding to phosphomannomutase (EC 5.4.2.8), which reversibly converts D-mannose 6-phosphate to α-D-mannose 1-phosphate, were identified; four ESTs were found in library I and three ESTs in library II.

The direct precursors for galactomannan biosynthesis, GDP-D-mannose and UDP-D-galactose, are formed by the actions of GDP mannose phosphorylase (EC 2.7.7.22) and UDP-galactose 4-epimerase (EC 5.1.3.2). *In vitro *experiments have shown that the relative concentrations of these precursors can affect the M:G ratio of the galactomannan polymer [5]. Of the three ESTs corresponding to GDP mannose phosphorylase, one was found in library I and two in library II. Two ESTs corresponding to UDP-galactose 4-epimerase were detected only in library I.

**Figure 4:**
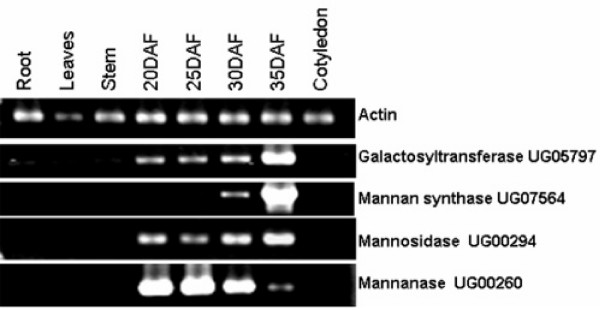
**RT-PCR analysis of genes involved in galactomannan biosynthesis and degradation**. RNA was isolated from seeds (20, 25, 30 and 35 DAF), roots, leaves, stems and cotyledons.

Two tightly membrane-bound glycosyltransferases together catalyze the formation of galactomannans. GDP-mannose-dependent mannosyltransferase transfers mannose residues to the end of the growing linear (1→4) β-linked mannose backbone of the galactomannan polymer [5,6,20]. Simultaneously, UDP-galactose-dependent galactosyltransferase transfers a galactose residue through a (1→6) α-linkage to a mannose at or near the nonreducing end of the growing mannan chain [5,6]. Importantly, galactose can not be transferred to preformed mannose chains [4]. The activities of the two transferases increase in parallel during the period of galactomannan synthesis, such that the M:G ratio in the polymer remains constant [4-6]. UG07564, represented as a single EST in library I, was 100% identical to a recently described guar β-mannan synthase sequence [14]. RT-PCR analysis with RNA from guar roots, leaves, stems, cotyledons and different development stages of seeds, revealed that this gene was only expressed in seeds, with maximum transcript accumulation at 35 DAF (Figure 4). In a previous study [14] 10 ESTs corresponding to β-mannan synthase were found in a library derived from guar endosperm at 25 DAF. The low frequency of β-mannan synthase ESTs in our work may be due to the fact that our libraries were constructed from whole seed tissues.

It is not known how many isoforms of β-mannan synthase and galactosyl transferase are involved in galactomannan biosynthesis in guar. To highlight additional candidate β-mannan synthase genes, we considered all ESTs which show similarity to glycosyl transferase family 2 members, which are able to transfer GDP-mannose to a range of substrates. By this criterion, four additional ESTs representing putative β-mannan synthase were found, three from library I and one from library II (Table 1).

UDP-galactose-dependent galactosyltransferase belongs to glycosyl transferase family 34 [36]. Two ESTs corresponding to galactosyltransferase were detected in our EST database; UG05797, from library II, showed 100% identity to a guar galactosyltransferase sequence available in GenBank, whereas UG03477, also from library II, showed 62% similarity to a galactosyltransferase from rice (*Oryza sativa*) (Table 1). RT-PCR analysis of different guar tissues showed the presence of UG03477 transcripts only in seeds, with maximal accumulation at 35 DAF (Figure 4), consistent with an involvement of this gene in galactomannan biosynthesis.

*Hydrolysis *– Three enzymes are involved in the hydrolysis of galactomannans during seed germination: β-mannosidase, which hydrolyses the oligomannans released by prior endo β-mannanase activity; β-mannanase, which cleaves the mannan backbone; and α-galactosidase which concomitantly removes the galactose side-chain units [37]. Galactomannan hydrolases were the most abundant class of ESTs involved in galactomannan metabolism in the seed EST libraries. Of the five genes annotated as β-mannanase, UG00260 and UG00259 were highly represented in library I, by 10 and 12 ESTs respectively. RT-PCR analysis showed the highest expression level for UG00260 to be at 20–25 DAF (Figure 4). Thus, β-mannanases are actively transcribed during early seed development in guar. Schroder et al (2006) recently demonstrated that a tomato endo-β-mannanase can carry out a transglycosylation in the presence of mannan-derived oligosacchrides [31]. This observation may support our findings of high steady-state levels of β-mannanase transcripts in developing seeds.

Of the three β-mannosidase genes detected only in library II, UG00294 was the most highly expressed, being represented by 14 ESTs. RT-PCR confirmed elevated transcript levels for this gene at 30–35 DAF (Figure 4). α-Galactosidase appeared to be less highly expressed; from four identified unigenes, only three ESTs were present in library I and one in library II (Table 1). Early transcriptional activation of galactomannan hydrolyzing enzymes is consistent with seed biology. Upon imbibition, pre-formed enzymes present in the aleurone layer are secreted to mobilize the stored reserves during seed germination [38]. Nevertheless, it does raise the question of whether degradative enzymes are ever in proximity with galactomannan during its biosynthesis, such that overall chain length or composition is modified prior to storage.

### Seed storage proteins

Seed storage proteins (SSPs) are a set of proteins that accumulate to high levels in seeds during the late stages of development. During germination, SSPs are degraded and the resulting amino acids are utilized by the developing seedlings as a nutritional source [39,40]. In mature guar seeds, protein accounts for 23–31% of seed dry weight [25].

**Figure 5:**
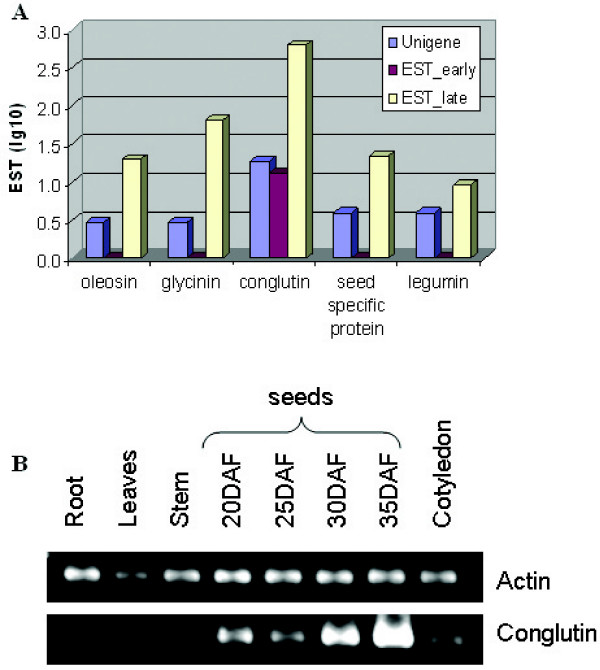
**Expression during seed development**. (A) EST counts for seed storage proteins in the "early" and "late" guar cDNA libraries. EST numbers were log base10 transformed, which reduce the effects of outliers, for better visualization the EST level of seed storage proteins in "early" and "late" seed libraries. (B) RT-PCR analysis of guar conglutin (UG00199) expression. RNA was isolated from roots, leaves, stems, seeds (20, 25, 30 and 35 DAF) and cotyledons.

Five classes of unigenes representing seed-specific proteins were identified in both guar libraries and showed similarities to oleosin, glycinin, conglutin, "seed specific protein," and legumin. All except glycinin did not pass the "secure" assignment threshold of S ≥ 200 (Figure 5A, Table 2). Usually, SSP sequences predominate in cDNA libraries derived from seeds [16]. The SSPs were not subtracted from the libraries described here. A single SSP, UG00199, represented the largest class of clones, with 602 ESTs in library II and comprising 3.7% of the total ESTs from both libraries. The predicted translation product of this gene contained 146 amino acids and showed 51% amino acid identity to the delta-conglutin seed storage protein precursor from *Lupinus albus*. Conglutin delta is related to the 2S super-family of storage proteins [41]. 2S storage proteins are widely distributed in dicot seeds, including the economically important genera *Brassica *[42] and *Pisum *[43], as well as the model plant *Arabidopsis *[44]. The family is characterized by low molecular weight proteins that contain relatively high levels of cysteine and glutamine.

**Table 2:** Seed specific proteins

**Unigene ID**	**Early**	**Late**	**NR Top Hit**	**NR Top Hit Description**	**Score**
GUAR_UG_00232	0	6	AAM46778	oleosin [Theobroma cacao]	4e-029
GUAR_UG_00334	0	11	AAU21499	oleosin 1 [Arachis hypogaea]	9e-012
GUAR_UG_00695	0	3	AAZ20277	oleosin 2 [Arachis hypogaea]	0.022
					
GUAR_UG_00201	0	20	AAP37971	seed specific protein [Brassica napus]	1e-015
GUAR_UG_05274	1	0	AAP37971	seed specific protein [Brassica napus]	1e-016
GUAR_UG_05457	0	1	AAP37971	seed specific protein [Brassica napus]	1e-014
GUAR_UG_06730	0	1	AAP37971	seed specific protein [Brassica napus]	1e-012
					
GUAR_UG_00136	0	43	CAA60533	glycinin [Glycine soja]	2e-059
GUAR_UG_07275	0	1	BAC55937	glycinin A1bB2-445 [Glycine max]	2e-061
GUAR_UG_00164	0	22	CAA33217	glycinin subunit G3 [Glycine max]	1e-049
					
GUAR_UG_03863	1	0	CAA37598	conglutin delta [Lupinus angustifolius]	0.045
GUAR_UG_06076	0	1	CAA37598	conglutin delta [Lupinus angustifolius]	2e-005
GUAR_UG_00199	12	602	CAJ43922	conglutin delta seed [Lupinus albus]	3e-025
GUAR_UG_00205	0	3	CAJ43922	conglutin delta seed [Lupinus albus]	1e-004
GUAR_UG_00417	0	7	CAJ43922	conglutin delta seed [Lupinus albus]	2e-006
GUAR_UG_05291	0	1	CAJ43922	conglutin delta seed [Lupinus albus]	7e-013
GUAR_UG_05432	0	1	CAJ43922	conglutin delta seed [Lupinus albus]	5e-012
GUAR_UG_05435	0	1	CAJ43922	conglutin delta seed [Lupinus albus]	3e-017
GUAR_UG_05535	0	1	CAJ43922	conglutin delta seed [Lupinus albus]	2e-016
GUAR_UG_05588	0	1	CAJ43922	conglutin delta seed [Lupinus albus]	4e-014
GUAR_UG_05592	0	1	CAJ43922	conglutin delta seed [Lupinus albus]	3e-016
GUAR_UG_05865	0	1	CAJ43922	conglutin delta seed [Lupinus albus]	2e-021
GUAR_UG_06252	0	1	CAJ43922	conglutin delta seed [Lupinus albus]	8e-010
GUAR_UG_06353	0	1	CAJ43922	conglutin delta seed [Lupinus albus]	2e-012
GUAR_UG_06800	0	1	CAJ43922	conglutin delta seed [Lupinus albus]	5e-019
GUAR_UG_07215	0	1	CAJ43922	conglutin delta seed [Lupinus albus]	1e-005
GUAR_UG_07438	0	1	CAJ43922	conglutin delta seed [Lupinus albus]	3e-009
GUAR_UG_07609	0	1	CAJ43922	conglutin delta seed [Lupinus albus]	4e-019
GUAR_UG_07626	0	1	CAJ43922	conglutin delta seed [Lupinus albus]	2e-004
					
GUAR_UG_00183	0	6	CAA30067	legumin [Pisum sativum]	7e-004
GUAR_UG_07620	0	1	CAA30068	legumin [Pisum sativum]	8e-004
GUAR_UG_05308	0	1	CAA83674	legumin B [Vicia sativa]	6e-016
GUAR_UG_05315	0	1	CAA83674	legumin B [Vicia sativa]	6e-016

**Figure 6:**
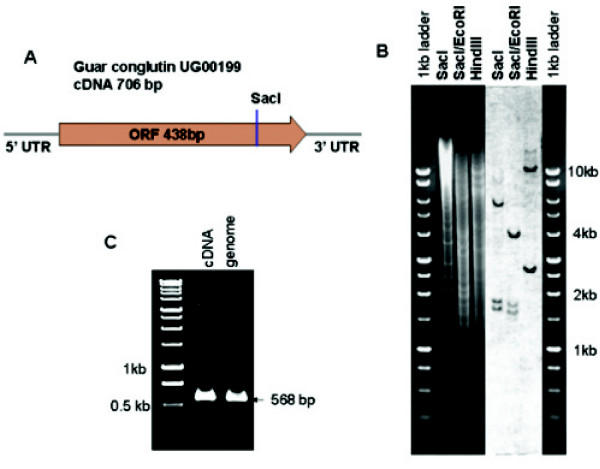
**Genomic organization of the guar conglutin gene**. (A) Schematic diagram of the guar conglutin cDNA. (B) DNA gel blot analysis of guar conglutin. Genomic DNA was digested with SacI, SacI/EcoRI and HindIII restriction endonucleases. The first and last lanes represent 1 kb ladder molecular weight markers, the second through fourth lanes show guar genomic DNA digested with SacI, SacI/EcoRI and HindIII, respectively; the fifth through seventh lanes show the blot hybridized with the conglutin probe.(C). PCR analysis of the guar conglutin gene from cDNA and genomic DNA templates.

RT-PCR analysis of guar conglutin transcripts showed maximal expression level in seeds at 35 DAF, and a low but detectable level of expression in cotyledons (Figure 5B). Amplification of conglutin from genomic DNA showed the PCR product to be the same size as the cDNA, indicating that the gene lacks introns (Figure 6C). DNA gel blot analysis of the conglutin, which contains a SacI restriction site in its open reading frame, revealed a low copy number in guar genomic DNA (Figure 6A–B).

## Conclusion

We present information on a large data set of ESTs from two developmental "windows" of guar seeds, and provide a preliminary analysis of this resource. Based on our analysis, it is clear that widely differing sets of genes are activated at the "early" and "late" developmental stages. Approximately 27% of the clones in the seed dataset correspond to novel proteins. The functional ontologies with the largest numbers of ESTs were signal transduction, carbohydrate metabolism, translation and protein processing. Overall the "late" cDNA library contained fewer genes in each functional category, except for storage proteins, hormonally-induced and pathogen-stress induced genes. Two major products accumulate in mature guar seeds: galactomannan and protein representing 26–32% and 23–31% of the seed dry weight, respectively [25]. Guar unigenes involved in galactomannan metabolism were identified. Among the seed storage proteins the most abundant contig represented a conglutin.

## Methods

### Plant materials

Guar (*Cyamopsis tetragonoloba) *plants, cultivar HES 1401 (now known as Monument, Plant Variety Protection Number: 200400301), were used in this study. This cultivar grows up to 11 dm tall and has the greatest amount of soluble dietary fiber in the seeds [25]. Individual plants were grown in 2 gallon pots containing 75% soil (Metro Mix 350, Sun Gro Horticulture, Bellevue, WA) and 25% sand at a temperature of 26°C/22°C (day/night). Plants were fertilized at time of watering using a commercial fertilizer mix (Peters Professional 20-10-20 (N-P-K) General Purpose, The Scotts Company, Marysville, Ohio).

### Construction of guar cDNA libraries

Seeds from guar cultivar HES 1401 were harvested 15, 20, 25, 30, 35, and 40 days after flowering (DAF). Total RNA was extracted from 200–500 mg of ground tissue from the six different seed stages collected from 10 plants using TRI Reagent (Molecular Research Center, Inc. Cincinnati, OH) following the manufacturer's recommendations. Poly A+ RNA was isolated using an Oligotex mRNA Mini Kit (Qiagen, Los Angeles, CA). cDNA was prepared from polyA+ enriched, pooled samples of equivalent amounts of total RNA from each time point. Two cDNA libraries were generated: an "early" seed library (15, 20, and 25 DAF, library I), and a "late" seed library (30, 35, and 40 DAF, library II). The cDNA was directionally ligated into the Uni-Zap XR vector (Stratagene, Los Angeles, CA) and packaged using Gigapack III Gold packaging extracts. Phagemids containing cDNA inserts were *in vivo *excised from the recombinant Uni-ZAP XR vector using ExAssist helper phage and the *E. coli *strain XL1-Blue MRF' (Stratagene, Los Angeles, CA). Excised plasmids were plated using SOLR cells (Stratagene, Los Angeles, CA).

### EST processing, assembly and gene annotation

Plasmid preparations were made using a Beckman Biomek 2000 robot following standard protocols. Average insert size (1–1.5 kb) was evaluated by agarose gel electrophoresis. cDNA clones were sequenced (single pass, 5'-end sequencing) using an Applied Biosystems 3730 sequencer. Base calling and conversion of binary trace files (.ab1) to human readable text files (.phd.1 and .seq) was completed using Applied Biosystems Sequence Analysis 5.1 program, which essentially is based on Phred [45]. Raw sequences were screened and cleaned with NCGR's X Genome Initiative (XGI) program [46], which removed the low quality (N-rich) reads, poly-A and low-complexity regions, vector and primer oligonucleotide sequences. 16,476 quality EST sequences with a minimal length of 50 bp were saved for downstream analysis. These include 8,163 from library I and 8,313 from library II. EST sequences were further clustered and assembled into consensus (unigenes) with TIGR Assembler [47] using its default parameter settings (at least 40 bp overlap with 94% identity). The assembly process generated 7,694 consensus sequences, including 1,695 contigs and 5,999 singletons. BLAST search against the most current version (January 24, 2006) of NCBI non-redundant protein database (NR) was performed with the Personal BLAST Navigator (PLAN) system [48]. Annotations, including gene ontology (GO) annotation [39], on each query with the top hit that passed filters e-value ≤ 0.1 and score S' ≥ 40 were further analyzed. The BLASTX search adopted the commonly-used BLOSUM62 scoring matrix. The use of both e-value and score S' [21] filters ensures that only satisfactorily precise (low e-value) and relatively long (high score) alignments are studied [49].

### Microscopy

Guar seeds from 25 and 40 DAF were frozen in liquid nitrogen and sectioned to 15 micron by a microtome in a Leica CM1850 cryostat. Sections were stained with toluidine blue (0.05% w/v) to reveal non-neutral cell wall polysaccharides.

### RT-PCR

One μg of total RNA was used in a first strand synthesis using SuperScript III Reverse Transcriptase (Invitrogen Life Technologies, Chicago, IL) in a 20 μl reaction with oligo-dT primers according to the manufacturer's protocol. Two μl of the first strand reaction was used for PCR with Takara Ex Taq (Fisher Scientific Company, Palatine, IL) according to the manufacturer's protocol. PCR products were analyzed on an agarose gel. The sequences of primers used in RT-PCR experiments are listed in Table 3.

**Table 3:** DNA sequence of PCR primers used in the present work

**Gene Name**	**Forward primer**	**Reverse primer**
Actin	GGCTGGATTTGCTGGAGATGATGC	CAATTTCTCGCTCTGCTGAGGTGG
Galactosyl transferase UG05797	GGGACGAGAAGCGTAAGG	CTCCTCCTCAACCCTTCC
Mannan synthase UG07564	CAAGTCACTAGTCCATCCTGC	TACAGTTCTATGCTTATGGATAGC
Mannosidase UG00294	GCTATATTCTCCGTGACATCCAG	CACAAAGCGCCAAGTTAAACTCG
Mannanase UG00260	GGCTCTTCAACAAGCTTCTAACC	GGTCCACTTTGCTTGAGTTTGGC
Conglutin UG00199	CATTACACTCCTACAGAAACGGTGAG	AAGGCAACAAAGCACACTCTAAGTGC

### Isolation of genomic DNA and DNA gel blot hybridization

Young leaves from guar cultivar HES 1401 were frozen and ground in liquid nitrogen. Genomic DNA was extracted from 0.5 g ground tissue using Plant DNAZOL Reagent (Invitrogen Life Technologies, Chicago, IL) according to the manufacturer's protocol.

Ten μg of genomic DNA was digested with SacI, SacI/EcoRI or Hind III and loaded on a 0.8% agarose gel. The gel was capillary blotted to nylon Hybond-N+ membrane (Amersham Pharmacia Biotech, Pittsburgh, PA). The blot was hybridized and signal detected using ECL direct nucleic acid labelling and detection systems (Amersham Pharmacia) according to the manufacturer's protocol. Probe was synthesized by PCR using primers complementary to the conglutin gene listed in Table 3.

## Abbreviations

DAF – days after flowering

UG – unigene

## Authors' contributions

MN performed cDNA library and RT-PCR analyses, DNA gel blot analysis of the guar conglutin gene, and wrote the first draft of the manuscript. IT-J generated the cDNA libraries and assisted in performing DNA sequence analysis. SA maintained and harvested plant materials and performed preliminary DNA sequence and RT-PCR analyses. JH and PZ performed DNA sequence and statistical analyses. RAD and GDM conceived of the study, directed the experimentation, and assisted in the preparation of the manuscript. All authors read and approved the final manuscript.

## Supplementary Material

Additional file 1 Guar unigene analysis. The data provided represent the analysis of the "Early" and "Late" guar seed library unigenes. Click here for file
